# Executive Functions and Special Educational Needs and Their Relationship with School-Age Learning Difficulties

**DOI:** 10.3390/children11111398

**Published:** 2024-11-19

**Authors:** Juan Manuel Núñez, Ana Soto-Rubio, Marián Pérez-Marín

**Affiliations:** 1 Faculty of Psychology and Speech Therapy, Universitat de València, 46010 Valencia, Spain; nujuanma@alumni.uv.es; 2 Department of Developmental and Education Psychology, Universitat de València, 46010 Valencia, Spain; ana.soto@uv.es; 3 Department of Personality, Assessment and Psychological Treatments, Universitat de València, 46010 Valencia, Spain

**Keywords:** executive functions, learning difficulties, special educational needs, neurodevelopmental disorder

## Abstract

Background/Objectives: The relationship between executive functions, special educational needs (SEN), and learning difficulties in school-aged children is critical for developing effective educational interventions. This study explores the connection between executive functions and SEN in primary school students, examining differences in executive function profiles between those with and without SEN and their impact on learning difficulties. Methods: In total, 123 primary school students aged 6 to 12 and their teachers and parents participated in this study. The Behavior Rating Inventory of Executive Function (BRIEF-2) and the Prediscal test were used to assess difficulties in reading and mathematics, and sociodemographic and clinical data were collected through ad hoc records. Results: The results indicated that students with SEN exhibited significantly more affected executive function profiles compared to their peers without SEN in both family and school contexts, highlighting areas such as cognitive flexibility, initiative, working memory, planning and organisation, task supervision, and material organisation. Additionally, significant negative correlations were found between executive functions and performance in reading and mathematics, suggesting that deficits in executive functions are strongly associated with SEN. Conclusions: These findings underscore the critical role of executive functions in understanding and addressing SEN and learning difficulties, emphasising the need for comprehensive assessment programmes and early intervention targeting executive function deficits to support the academic and overall development of students with SEN.

## 1. Introduction

Most societies today recognise education as a fundamental driver of human development and social change [1]. For this reason, they make it a priority on their agendas, taking into account that the school system responds to various needs beyond the curriculum, such as the comprehensive development of students and the promotion of a fairer society [2].

The association between health and education is well established [3]. Education has been described as the most important modifiable social determinant of health [4]. As stated in the Incheon Declaration 2030, promoted by UNESCO to generate a global approach, education is recognised as the most effective means of achieving gender equality, enabling the full social and political participation of girls and women and empowering them economically, and also, as one of the most efficient ways of improving people’s health and ensuring that the benefits are passed on to future generations. This declaration, therefore, urges education systems to ensure access to quality, inclusive, and equitable education, safeguarding optimal development and well-being in childhood and adolescence [5]. Likewise, the World Health Organisation (WHO), in its General Programme of Work and the Global Education Agenda 2030, sets out access to quality educational care as a priority for healthy human development [6].

According to Francisco, Hartman, and Wang [7], in this context, in which it is a priority to safeguard the fundamental human right to quality education and ensure comprehensive health and optimal development in childhood and adolescence, children with special educational needs (SEN) represent a group at risk that may not see these fundamental needs met.

According to UNESCO [8], a student is considered to have SEN when, for a wide variety of reasons, he/she shows more significant difficulties than the rest of his/her peers in accessing the learning that corresponds to his/her age or grade and requires extraordinary and specialised support to compensate for these difficulties, which, if not provided, limits his/her learning and development opportunities. SEN, therefore, cover a range of needs, including physical, sensory, mental, and cognitive disabilities and learning, emotional, and social difficulties [9]. This SEN approach highlights the need for a specific educational response to compensate for the significant difficulties that some students experience in accessing the curriculum, especially when associated with neurodevelopmental disorders, such as autism or attention deficit. These disorders affect academic performance and require personalised interventions to ensure inclusion in the school environment [10]. The Organic Law for the Improvement of Educational Quality (LOMCE) [11] considers students with SEN to be those with a wide range of conditions that may pose a barrier to the student’s academic, social, and comprehensive development. In Spain, data from the Ministry of Education and Culture for the 2022–2023 school year indicate an incidence of pupils with SEN of 12%. Of them, 27.2% correspond to students with special educational needs, where the support is associated with some disability or severe disorder, and the remaining (72.8%) correspond to students with other specific educational support needs.

At the same time, data indicate an increase of 42.1% in the school population with SEN in Spain over the past decade, underlining the importance of addressing the needs of this group not only to guarantee access to quality inclusive education but also to ensure their physical, psychological and social well-being [12]. This increase in SEN in childhood may be related to several factors, such as greater awareness and recognition of transient or permanent conditions that potentially affect learning, greater social expectations and demands on education, and progress in detecting and diagnosing conditions that affect children’s development [2].

The social impact of the increase in students with SEN is not only an educational and potentially health-related challenge [13] but also an economic one since it can represent a high cost for the State. Thus, for example, the educational interventions and expenses associated with the care of students with SEN can be up to four times the value of those estimated for typically developing students [14]. In addition to the clear benefit in the prognosis of students with SEN through early detection and care strategies [15], the social and economic consequences highlight the importance of these [16].

SEN associated with disability or severe impairment often involves neurodevelopmental disorders (NDDs). NDDs imply a common category to group a broad and heterogeneous set of conditions that originate in some form of early, significant, and persistent disruption of the dynamic processes involved in brain development, inducing chronic deficits in functioning and adaptive behaviour [17].

**Figure 1:**
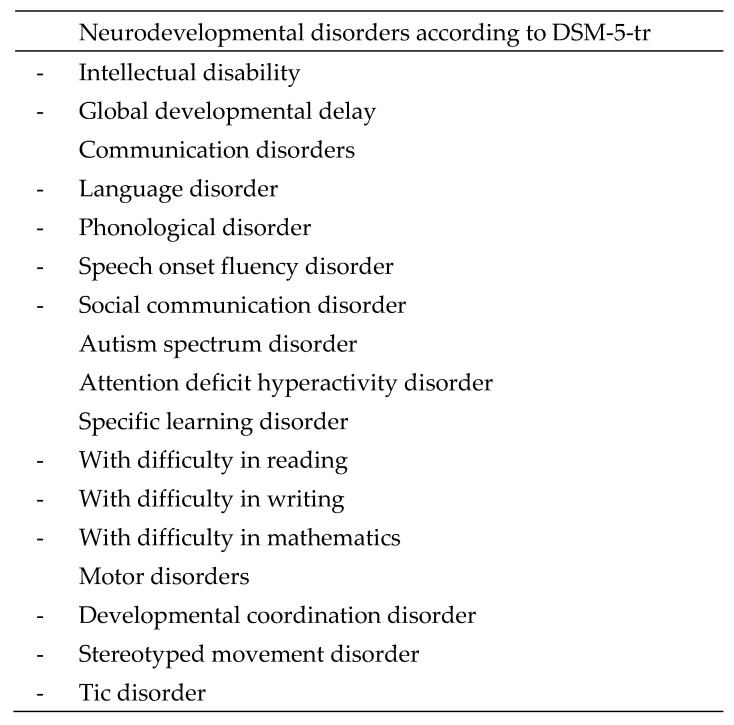
Neurodevelopmental disorders according to DSM-5-TR.

NDDs share the following characteristics: their origin is multifactorial, they begin in childhood, they hinder the acquisition of functions in several areas of development, they are more prevalent in males, they have a high comorbidity, and they present a chronic course. According to the Diagnostic and Statistical Manual of Mental Disorders (DSM-5-TR) [18], NDDs include several diagnoses (see Figure 1).

Despite the great symptomatic and etiological variety of neurodevelopmental disorders, all of them have in common a significant impairment of executive functions [19]. These functions are a meeting point for a wide variety of diagnoses, which is why a proper understanding of them is vital for conceptualising and approaching special educational needs associated with neurodevelopment.

Multiple definitions and theoretical models attempt to clarify the nature of executive functions (EFs). However, it remains one of the psychological constructs with the greatest need for study due to the need for more consensus regarding the term [20]. Executive functions are defined as a set of skills that are involved in the generation, monitoring, regulation, execution, and readjustment of appropriate behaviours to achieve complex goals, especially those that are novel for the individual and require a creative solution [21]. EF is often a synonym for the higher-order cognitive abilities needed to examine and achieve a goal. These functions enable us to understand complex situations or abstract concepts, solve new problems, plan our next behaviours, and manage relationships.

The development of EFs is a gradual process that extends through childhood and adolescence, with continuous improvement into early adulthood. These higher cognitive skills are essential for regulating behaviour and flexibly applying psychological processes and are based on distributed neural networks, most notably the prefrontal cortex, a region implicated in the deficits observed in neurodevelopmental disorders [22]. Regarding its normative development, a set of research indicates that basic executive functions emerge in early stages and show a certain structural consistency throughout childhood, which suggests a certain homogeneity in its neurological basis and basic functionality [23].

Likewise, EFs are essential for learning, problem-solving and adaptation to new situations. However, in neurodevelopmental disorders such as attention deficit hyperactivity disorder (ADHD), autism spectrum disorder (ASD), and specific learning difficulties (SLDs), these processes may be compromised, leading to additional difficulties in infants’ development [24]. For example, in ADHD, difficulties are observed in inhibiting impulsive responses and working memory, affecting attention span and task completion [25]. Similarly, in ASD, cognitive rigidity may hinder adaptation to new social situations [26].

Difficulties in executive functions, such as those mentioned above, can significantly impact the daily lives of schoolchildren and their future adult lives. In this sense, they may have problems with school skills and learning in general [27], affecting the academic environment in childhood and adolescence, with repercussions on their curricular performance and overall development. Later on, with their incorporation into adult life, due to these planning and organisation problems, they may have more difficulties when it comes to maintaining stable employment and meeting deadlines. Socially, difficulties in executive functions may make it difficult to establish interpersonal relationships and understand the emotions of others [28].

On the other hand, within SEN, we also find specific learning difficulties or developmental learning disabilities (SLDs), such as dyslexia, dyscalculia, and dysgraphia. SEN, as well as the learning process itself, are intrinsically linked to the development of executive functions. These neurodevelopmental disorders affect the acquisition of basic academic skills and can further hinder the learning process. The treatment of ASD involves a combination of educational and therapeutic interventions that address both the specific learning difficulties and the underlying executive function deficits. Executive function training may include strategies designed to improve attention, working memory, and planning, which helps individuals overcome learning difficulties and improve their overall quality of life [29].

In summary, understanding the relationship between executive functions, the special educational needs present in neurodevelopmental disorders, and their link to specific learning difficulties is fundamental to developing effective school interventions. By better understanding and addressing, more specifically, the deficits in executive functions, it will be possible to assess and intervene more accurately in students with special educational needs and to develop more comprehensive early detection programmes, promoting the overall development of those affected.

This work aims to study the link between executive functions, special educational needs, and learning difficulties in primary school pupils. More specifically, the aim is to (a) describe the profile of executive functions in primary school pupils and (b) analyse the differences between pupils with and without special educational needs in terms of executive functions.

## 2. Materials and Methods

### 2.1. Procedure and Sample

The study sample consisted of 123 primary school students attending mainstream schools in Castellón, Spain. There were 123 schoolchildren aged 6–12 ($\bar{X}$ = 8.61; SD = 1.8), of whom 53.6% were male, 3.2% had a medical diagnosis, and 11.2% had a psychological diagnosis. According to the teacher report, 31% (n = 38) of students presented SEN. The data were obtained directly from the students and complemented by information from their teachers and parents. The inclusion criteria were as follows:

(a) Children between 6 and 12 years of age in primary school, with and without SEN.
(b) If the child has special educational needs, he/she must have the necessary cognitive capacity to carry it out.
(c) The assessment had to be completed by the child himself/herself and, preferably, also by his/her parent and guardian. In order to diagnose SEN in children, the school report on adaptation measures for children with SEN was used as a criterion.

This study was approved by the ethics committee of the University of Valencia (reference UV-INV_ETICA-3119648) and the relevant permissions for the evaluation of primary school children from the Department of Education of the Valencian Community. It also followed the guidelines of the Law on Personal Data Protection (LOPD) 3/2018, of 5 December.

### 2.2. Instruments

Behavior Rating Inventory of Executive Function (BRIEF-2) [30]: It assesses the executive functions of schoolchildren between 5 and 18 years old according to school and family reports. Each report comprises 63 items with three response options (never, sometimes, and frequently). It includes nine executive functions (inhibition, self-monitoring, flexibility, emotional control, initiative, working memory, planning and organisation, task monitoring, and organisation of materials), three regulation scales (cognitive, behavioural, and emotional), and a global index of executive functions.

Screening for reading and mathematics difficulties (Prediscal) [31]: This is a test to detect the risk of learning difficulties. It is a self-applied test that can be carried out by schoolchildren from second to sixth grade of primary school. It consists of three tests that correspond to the three scales obtained: reading fluency (47 items), mathematical fluency (63 items), and calculation (45 items).

Ad hoc registers: Ad hoc registers were drawn up to obtain sociodemographic and clinical data on the children, school guardians, and parents. Specifically, the variables collected were the age and sex of the participants, type of participant (child, school guardian, or parent), academic year of the child, medical and/or psychological diagnosis of the child, level of schooling, employment status and marital status of the parent, and type of specialised care received by the children in both school and home contexts. In order to diagnose SEN in children, the school report on adaptation measures for children with SEN was used as a criterion.

### 2.3. Variables

The independent variables include participants’ age, gender, and special educational needs (SEN) status, classified into two categories: “Has SEN” and “Does not have SEN”.

The dependent variables are divided into two groups. The first group corresponds to executive functions, assessed by means of the BRIEF-2 questionnaire, which measures the following components: inhibition (impulse control and ability to stop automatic or inappropriate responses); self-monitoring (ability to self-evaluate and self-regulate behaviour); flexibility (adaptation to change and ability to switch between different tasks or thoughts); emotional control (regulation of emotional responses in an appropriate manner); initiative (ability to initiate activities or tasks autonomously); working memory (ability to retain and manipulate information temporarily for complex tasks); planning and organisation (development of strategies to perform tasks in a structured and organised manner); task monitoring (ability to remain attentive and aware of progress on a task); and organisation of materials (maintaining order in personal materials and belongings).

In addition, composite indices are included: behavioural regulation index (assessment of the ability to control impulses and behaviours); emotional regulation index (measurement of the ability to regulate emotional responses); cognitive regulation index (assessment of the ability to manage and regulate cognitive processes); and global index of executive function (general index that summarises the functioning of executive functions).

The second group of dependent variables is related to academic performance, assessed through the PredisCal scales, which cover the following areas: reading fluency (ability to read quickly and accurately); mathematical fluency (speed and accuracy in performing basic mathematical calculations); and calculation (competence in performing more complex mathematical operations).

## 3. Procedure

Data were collected on schoolchildren by their teachers, parents, and themselves at a single point in time. The schoolchildren and their parents came from the same schools in the province of Castellón in Spain, all of which were ordinary schools.

Training was carried out in the schools to train the tutors and counsellors regarding the completion and collection of data. After this, and after receiving the informed consent of the participating families and training them too, dates were agreed upon for the sample collection in the schools, both for the test to be completed by the schoolchildren and for the evaluation of the families and of the school tutors.

Four public and private educational centres in the Valencian Community were randomly selected, and an e-mail was sent requesting their participation in this study. Subsequently, a personal interview was arranged with those schools that showed interest in order to provide more detailed information on the aim of the research and, where appropriate, to confirm their participation. After obtaining institutional permission, the evaluation protocol was planned. This took place collectively during a tutoring session in the last term of 2022. After obtaining informed consent, the parents’ questionnaires were sent out on the same day and collected within a week. Of the 300 students, 123 participated.

### Statistical Analysis

The study of mean differences was carried out using a categorical independent variable, in which the criterion variable was the presence or absence of special educational needs (SEN), categorised into two groups: with SEN and without SEN. Differences between these groups were evaluated using Student’s t-test for independent samples. To test for homogeneity of variances, Levene’s test was applied, and the effect size was calculated using Cohen’s d in the cases in which the means of the independent samples were compared.

The dependent variables analysed in the study were executive functions and academic performance. In addition, in order to explore possible relationships between these dependent variables and other variables in the study, Pearson’s correlation coefficient was used. All statistical analyses were performed using SPSS version 29.0 software.

## 4. Results

There were 123 schoolchildren aged 6–12 ($\bar{X}$ = 8.61; SD = 1.8), of whom 53.6% were male, 3.2% had a medical diagnosis, and 11.2% had a psychological diagnosis. According to the teacher report, 31% of students presented SEN. Regarding specialised care at school, 7.2% received therapeutic pedagogy, and 8% received hearing and speech therapy. Regarding specialised care outside the school context, 60% did not receive it, and only 3.2% received psychological care (See Table 1).

A total of 88 parents participated, the most frequent profile being that of a mother (82.9%), with an age range of 29–57 ($\bar{X}$ = 41; SD = 5.25). Most of them were married (48%), had a level of education equivalent to a training course or baccalaureate (33.2%) or university studies (24.8%), and were in active employment, either with a permanent contract (28.8%) or self-employed (16.8%) (See Table 2).

Significant associations were found between most executive functions and learning, differing in some cases depending on the context of reporting (see Table 3). Several executive functions correlated negatively with the three measures of learning (mathematical fluency, reading fluency, and calculation) in family and school contexts: working memory, planning and organisation, task monitoring, cognitive regulation index, and overall executive function index. Flexibility correlated negatively with all three learning skills in the school context but only with calculation and mathematical fluency in the home context.

In the family report, only some executive functions showed a negative relationship in all cases: inhibition with calculation and mathematical fluency, initiative with the three learning scales, organisation of materials with calculation, and indices of behavioural and emotional regulation with calculation and mathematical fluency.

For simplicity in explaining the results, only data relating to significant results are presented in the following tables of results.

The comparison between pupils with and without SEN showed that, in general, the level of impairment of pupils with special educational needs was higher. It is important to remember that, in the results from this assessment test, as they are clinical measures, higher mean scores indicate greater impairment in executive function (See Table 4)

When observing the differences in executive functions between students with and without SEN, greater impairment was found in all the functions assessed in students with SEN, and this difference was generally observed in the two assessment contexts (family and school). Specifically, the executive functions with the worst level of development in students with SEN were cognitive flexibility, initiative, working memory, planning and organisation, task monitoring, and organisation of materials.

The same occurred with the indices of executive functions, such as the behavioural regulation index, the cognitive regulation index, and the global index of executive functions; the pupils with educational needs also presented greater clinical affectation than those without SEN.

Similarly, but only based on the assessments obtained from the tutor in the school context, higher scores were obtained for students with special educational needs in inhibition and the emotional control.

Regarding the level of learning assessed by Prediscal when comparing students with and without clinical impairment in executive functions, it was observed that, in all cases, the profile of students with clinical impairment in executive functions was worse in the three domains assessed: reading fluency, mathematical fluency, and calculation.

Generally, schoolchildren without clinical impairment performed better in all the areas assessed. Specifically, in reading fluency, the indices of working memory, planning, and task monitoring in the home environment, as well as the cognitive regulation index and global index of executive functions at school, showed significantly higher means in the non-clinically affected group (Table 5). Similarly, in mathematical fluency (Table 6), significant differences were observed in working memory, planning, task monitoring, and initiative in home and school settings.

The greatest impairment was observed in calculus (Table 7), where the differences between clinically and non-clinically impaired schoolchildren were even more pronounced. Executive functions related to initiative, working memory, and planning in home and school environments showed the greatest disparities. Specifically, schoolchildren without clinical executive function impairment had significantly higher means in all categories tested.

## 5. Discussion

Schoolchildren with special educational needs (SEN) present a profile of executive functions that is significantly more impaired compared to their peers without SEN. In both assessments, in the family and school contexts, students with SEN scored significantly higher on most executive functions, indicating a greater impairment in their performance. These differences were evident in critical functions such as cognitive flexibility, initiative, working memory, planning and organisation, task monitoring and organisation of materials. In addition, the indices of behavioural, emotional, and cognitive regulation reflected unfavourable results for schoolchildren with SEN. These findings are consistent with previous research suggesting that children with SEN experience greater executive function difficulties [32].

A significant negative correlation was also identified between the executive function profile and the presence of SEN, indicating that lower performance in these executive functions is associated with SEN. The executive functions that showed a higher degree of impairment in schoolchildren with SEN included working memory, planning and organisation, task monitoring, and cognitive flexibility. These results suggest that difficulties in executive functions may be an essential underlying factor in identifying and managing SEN, supporting the existing literature that emphasises the relationship between executive functions and academic performance [33].

While significant differences in the assessment of executive functions were documented between the family and school contexts, these differences were not uniform across all functions assessed. In the school context, assessments by guardians indicated greater impairment in specific functions such as inhibition and emotional control. In contrast, in the family context, parents reported greater difficulties in organising materials and emotional regulation. These findings underline the importance of considering multiple sources of information when assessing executive functions in children, given that different informants may observe and report different difficulties depending on the environment and the relationship with the child [34].

These findings have implications for educational and social settings. In the school environment, the early identification of executive function difficulties could facilitate the implementation of specific interventions to support the learning and adaptation of students with SEN. Considering each student’s particularities, personalising educational strategies can improve their academic performance and emotional well-being.

From a social perspective, it is essential to promote awareness of the importance of executive functions in children’s overall development. Training teachers and parents to identify and support the specific needs of these students can contribute to a more inclusive and supportive environment.

Although significant, this study has certain limitations that need to be considered. Firstly, assessing executive functions could benefit from incorporating more varied methods and conducting longitudinal follow-ups to observe changes over time.

Future research could investigate the specific strategies that schoolchildren with special educational needs (SEN) employ to manage challenges related to executive functions, as well as evaluate the effectiveness of interventions designed to enhance these skills across various settings. Additionally, further studies could explore diagnostic differences in executive function, deepening our understanding of how these functions influence children’s educational and social development.

## 6. Conclusions

The first objective of the present study was to analyse the differences in executive functions between students with and without special educational needs (SEN). The results obtained show that students with SEN present significant deficits in several executive functions compared to those without SEN. These differences highlight the importance of executive functions in academic and social development, since these abilities influence behavioural regulation, organisational capacity, and adaptation to the school context. The presence of these deficits can hinder the academic performance of students with SEN and limit their autonomy, underscoring the need to design educational interventions that address these specific areas.

The second objective of this study was to explore the relationship between executive functions and academic performance in students with and without SEN. The analyses carried out confirm the existence of a significant correlation between both variables, especially in those academic areas that require organisation, time management, and planning skills. This finding shows how the adequate development of executive functions can have a positive impact on the academic performance of students, facilitating better performance in the school environment. In this sense, the strengthening of executive functions could not only improve academic performance, but also contribute to better adaptation and emotional and social well-being of students.

In conclusion, the findings of this study highlight the importance of implementing educational interventions focused on the development of executive functions, especially in students with SEN. This approach would allow addressing academic difficulties and, at the same time, fostering skills that are essential for active and autonomous participation in society. Furthermore, these results provide relevant practical implications for the design of inclusive educational programmes that consider the diversity in students’ cognitive abilities and provide the necessary support for each profile. It is essential that further research continues to explore the effectiveness of specific interventions in this population, in order to move towards a more equitable and evidence-based educational approach.

## Figures and Tables

**Table 1:** Specialised care received outside the school context.

Type of Care	n	Percentage
Without attention	75	60%
Psychology	4	3.2%
Speech therapy	1	0.8%
Pedagogy	3	2.4%
Interdisciplinary	3	2.4%

**Table 2:** Sociodemographic variables of parents.

Variable	N	Percentage
Civil status		
Single	6	4.8%
Married	61	48.8%
Divorced	10	8.0%
Widower	2	1.6%
Living as a couple	7	5.6%
Employment situation		
Non-temporary contract	36	28.8%
Temporary contract	7	5.6%
Self-employed	21	16.8%
Official	6	4.8%
Unemployed	15	12.0%
Other	1	0.8%
Education level		
Without studies	3	2.4%
Mandatory studies	9	7.2%
Bachelor/Professional cycles	42	33.6%
University studies	31	24.8%
Post-university studies	1	0.8%

**Table 3:** Correlation between study variables.

	Calculation	Mathematic F.	Reading F.
Family context			
Inhibition	−0.278 *	−0.274 *	−0.052
Monitoring yourself	−0.195	−0.135	−0.089
Flexibility	−0.256 *	−0.257 *	−0.102
Emotional control	−0.169	−0.214	0.013
Initiative	−0.370 **	−0.351 **	−0.262 *
Working memory	−0.491 **	−0.462 **	−0.353 **
Planning and organisation	−0.334 **	−0.403 **	−0.294 *
Task supervision	−0.334 **	−0.352 **	−0.385 **
Material organisation	−0.246 *	−0.176	−0.197
Behavioural R.	−0.277 *	−0.249 *	−0.074
Emotional R.	−0.236 *	−0.269 *	−0.045
Cognitive R.	−0.411 **	−0.406 **	−0.336 **
Executive F.	−0.386 **	−0.389 **	−0.241 *
School context			
Inhibition	−0.032	−0.121	−0.139
Monitoring yourself	−0.026	−0.040	−0.081
Flexibility	−0.293 **	−0.213 *	−0.248 *
Emotional control	0.103	0.088	−0.010
Initiative	−0.295 **	−0.288 **	−0.279 **
Working memory	−0.367 **	−0.343 **	−0.343 **
Planning and organisation	−0.318 **	−0.331 **	−0.383 **
Task Supervision	−0.337 **	−0.255 *	−0.429 **
Material organisation	−0.106	−0.140	−0.203
Behavioural R.	−0.034	−0.090	−0.118
Emotional R.	−0.145	−0.100	−0.180
Cognitive R.	−0.331 **	−0.310 **	−0.371 **
Executive F.	−0.261 *	−0.258 *	−0.327 **

Note: * *p* ≤ 0.05; ** *p* ≤ 0.01, F. = Features; R. = Regulation.

**Table 4:** Comparison of means between students with SEN and without SEN, according to school and family report.

Variables	Without SEN (n = 85) (Mean; SD)	With SEN (n = 38) (Mean; SD)	*t*	*p*	*d*
Family Context					
Self-monitoring	(49.68; 8.83)	(55.92; 10.82)	−2.854	0.003	9.432
Flexibility	(51.56; 10.90)	(58.23; 12.55)	−2.484	0.007	11.395
Initiative	(51.56; 11.09)	(59.69; 13.86)	−2.949	0.002	11.936
Work memory	(50.75; 11.56)	(64.46; 12.70)	−4.769	<0.001	11.898
Planning and organisation	(52.43; 11.78)	(60.23; 12.96)	−2.772	0.003	12.126
Task monitoring	(50.78; 10.55)	(58.04; 9.97)	−3.008	0.002	10.393
Material organisation	(51.49; 10.50)	(56.73; 13.81)	−1.958	0.027	11.530
Behavioural regulation index	(50.77; 9.23)	(55.08; 10.73)	−1.918	0.029	9.680
Cognitive regulation index	(52.43; 10.56)	(56.19; 10.87)	−3.627	<0.001	12.303
Global index of executive functions	(51.95; 11.59)	(60.46; 11.89)	−3.139	0.001	11.679
School Context					
Initiative	(43.90; 6.87)	(56.73; 15.31)	−3.255	0.001	10.803
Self-monitoring	(46.63; 9.48)	(54.18; 14.44)	−2.886	0.003	11.106
Flexibility	(41.64; 4.93)	(55.85; 14.80)	−5.394	<0.001	8.891
Emotional control	(44.86; 3.97)	(50.24; 13.41)	−2.668	0.015	7.865
inhibition	(43.90; 6.87)	(56.73; 15.31)	−4.628	<0.001	9.991
Work memory	(43.88; 2.93)	(60.09; 14.69)	−6.248	<0.001	8.471
Planning and organisation	(42.95; 4.57)	(55.97; 12.35)	−5.896	<0.001	7.608
Task monitoring	(43.88; 2.93)	(57.91; 13.17)	−5.985	<0.001	9.097
Material organisation	(44.89; 4.74)	(54.79; 16.04)	−3.484	<0.001	9.405
Behavioural regulation index	(45.07; 8.67)	(54.67; 16.11)	−3.240	0.001	11.267
Emotional regulation index	(42.34; 4.43)	(53.27; 12.97)	−4.732	<0.001	7.338
Cognitive regulation index	(42.78; 4.51)	(58.45; 14.50)	−6.090	<0.001	8.587
Global index of executive functions	(42.20; 5.19)	(57.52; 13.37)	−6.387	<0.001	8.344

Note: SD = standard deviation; Cohen’s d: small TE ≈ 0.20; moderate TE ≈ 0.50; large TE ≈ 0.80.

**Table 5:** Comparison of means between schoolchildren with and without clinical impairment in executive functions in reading fluency according to Prediscal results.

Executive Function (Brief-2)	No Clinic Affection (Mean; SD)	Clinic Affection (Mean; SD)	t	*p*	d
Working memory FC	(n = 54) (54.83; 28.228)	(n = 16) (39.00; 30.98)	1.928	0.029	28.859
Planning and organisation FC	(n = 53) (54.15; 28.510)	(n = 18) (41.39; 30.40)	1.614	−0.056	28.988
Task supervision FC	(n = 61) (53.44; 29.008)	(n = 10) (35.50; 27.722)	1.823	0.036	28.843
Cognitive regulation index SC	(n = 83) (50.80; 31.164)	(n = 7) (27.29; 22.823)	1.948	0.027	30.667
Global index of executive functions SC	(n = 84) (50.31; 31.294)	(n = 7) (30.14; 21.513)	2.287	0.025	30.732

Note: SD = standard deviation; Cohen’s *d*: small TE ≈ 0.20; moderate TE ≈ 0.50; large TE ≈ 0.80; SC = School context; FC = family context.

**Table 6:** Comparison of means between schoolchildren with and without clinical impairment in executive functions in mathematical fluency according to Prediscal results.

Executive Function (Brief-2)	No Clinic Affection (n = 85) (Mean, SD)	Clinic Affection (n = 38) (Mean, SD)	*t*	*p*	*d*
Working memory FC	(n = 54) (47.43; 28.496)	(n = 16) (26.63; 22.003)	3.239	0.001	27.197
Planning and organisation FC	(n = 53) (46.09; 29.938)	(n = 18) (30.39; 19.980)	2.512	0.008	27.817
Task supervision FC	(n = 61) (44.92; 28.374)	(n = 10) (25; 23.551)	2.101	0.020	27.793
Initiative SC	(n = 82) (44.45; 23.78)	(n = 9) (23.78; 9.947)	4.441	<0.001	28.383
Working memory SC	(n = 83) (44.37; 29.369)	(n = 6) (21.17; 11.890)	3.983	0.001	28.655
Planning and organisation SC	(n = 85) (43.95; 29.270)	(n = 6) (20.50; 4.848)	6.269	<0.001	28.459

Note: SD = standard deviation; Cohen’s *d*: small TE ≈ 0.20; moderate TE ≈ 0.50; large TE ≈ 0.80; SC = School context; FC = family context.

**Table 7:** Comparison of means between schoolchildren with and without clinical impairment in executive functions in calculus according to Prediscal results.

Executive Function (Brief-2)	No Clinic Affection (n = 85) (Mean; SD)	Clinic Affection (n = 38) (Mean; SD)	*t*	*p*	*d*
Initiative FC	(n = 61) (45.56; 30.60)	(n = 10) (30.80; 16.55)	2.257	0.017	29.160
Working memory FC	(n = 54) (49.59; 29.137)	(n = 16) (22.63; 20.909)	3.441	<0.001	27.534
Planning and organisation FC	(n = 53) (48.72; 30.462)	(n = 18) (28.06; 19.633)	3.312	<0.001	28.183
Cognitive regulation index (SC)	(n = 54) (47.83; 30.744)	(n = 16) (28.56; 19.748)	2.978	0.011	28.638
Global index of executive functions (SC)	(n = 58) (47.28; 26.54)	(n = 13) (26.54; 17.510)	3.304	0.001	28.796
Initiative SC	(n = 82) (26.29; 29.766)	(n = 9) (23.22; 15.587)	3.752	<0.001	28.779
Working memory SC.	(n = 83) (46.20; 29.624)	(n = 6) (18.33; 12.176)	4.692	<0.001	28.907
Planning and organisation SC	(n = 85) (45.42; 29.736)	(n = 6) (24; 15.362)	3.038	0.008	29.117

Note: SD = standard deviation; Cohen’s *d*: small TE ≈ 0.20; moderate TE ≈ 0.50; large TE ≈ 0.80.; SC = School context; FC = family context.

## Data Availability

The data presented in this study are available on request from the corresponding author. They are not publicly available due to data privacy and confidentiality.

## References

[1] Steyn H., Vos D., Beer L.D. (2018). Education in Modern Society.

[2] López S.I.M., Valenzuela B.G.E. (2015). Niños y adolescentes con necesidades educativas especiales. Rev. Med. Clin. Las. Condes..

[3] Cutler D., Lleras-Muney A. (2006). Education and Health: Evaluating Theories and Evidence.

[4] Kolbe L.J. (2019). School health as a strategy to improve both public health and education. Annu. Rev. Public Health.

[5] Soto N.H. (2017). Reflexión teórica sobre la Declaración de Incheon Educación 2030 Hacia una educación inclusiva y equitativa de calidad y un aprendizaje a lo largo de la vida de todos. Rev. Educ. Inclus..

[6] World Health Organization (2015). General Programme of Work: Priorities Directions for, W.H.O..

[7] Francisco M.P.B., Hartman M., Wang Y. (2020). Inclusion and special education. Educ. Sci..

[8] UNESCO (2011). Inclusive Education: Addressing the Learning Needs of All Children.

[9] Castillo Retamal F., Cárcamo Garrido B., Aravena Calderón H., Valenzuela A., Pérez Farías T., Medel Tapia C., Quezada-Alcaíno J. (2021). Necesidades educativas especiales y educación física: Un análisis desde la propuesta curricular ministerial de Chile. Retos Digit.

[10] Van der Merwe M., Fourie J.V., Yoro A.J. (2020). Learning support strategies for learners with neurodevelopmental disorders: Perspectives of recently qualified teachers. Afr. J. Disabil..

[11] Ministerio de Educación, Cultura y Deporte (2020). Ley Orgánica Para la Mejora de la Calidad Educativa (LOMCE).

[12] Alcaraz-García S., Arnaiz-Sánchez P. (2020). La escolarización del alumnado con necesidades educativas especiales en España: Un estudio longitudinal. Rev. Colomb. Educ..

[13] Bertrán J.B. (2015). Psicopedagogía de la Diversidad en el Aula: Desafío a las Barreras en el Aprendizaje y la Participación.

[14] Rogge N., Janssen J. (2019). The economic costs of autism spectrum disorder: A literature review. J. Autism Dev. Disord..

[15] Vázquez-Villagrán L.L., Moo-Rivas C.D., Meléndez-Bautista E., Magriñá-Lizama J.S., Méndez-Domínguez N.I. (2017). Revisión del trastorno del espectro autista: Actualización del diagnóstico y tratamiento. Rev. Mex. Neurocien..

[16] Ainscow M. (2020). Promoting inclusion and equity in education: Lessons from international experiences. Nord. J. Stud. Educ. Policy.

[17] López I., Förster J. (2022). Trastornos del neurodesarrollo: Dónde estamos hoy y hacia dónde nos dirigimos. Rev. Med. Clin. Las. Condes..

[18] American Psychiatric Association (2022). Diagnostic and Statistical Manual of Mental Disorders.

[19] Mejía I.D.D., Rubiales J., Etchepareborda M.C., Bakker L., Zuluaga J.B. (2012). Intervención multimodal del TDAH: El papel coterapéutico de la familia. Rev. Arg. Clínica Psicol..

[20] Tirapu Ustárroz J., Cordero Andrés P., Bausela Herreras E. (2018). Funciones ejecutivas en población infantil: Propuesta de una clarificación conceptual e integradora basada en resultados de análisis factoriales. Cuad. Neuropsicol-Panam. J. Neuropsychol..

[21] Ustárroz J.T., Molina A.G., Lario P.L., García A.V., Lago M.R., Ustárroz J.T., Molina A.G., Lario P.L., García A.V. (2012). Corteza prefrontal, funciones ejecutivas y regulación de la conducta. Neuropsicología de la Corteza Prefrontal y las Funciones Ejecutivas.

[22] Friedman N.P., Robbins T.W. (2022). The role of prefrontal cortex in cognitive control and executive function. Neuropsychopharmacology.

[23] Moriguchi Y., Chevalier N., Zelazo P.D. (2016). Development of executive function during childhood. Front. Psychol..

[24] Crisci G., Caviola S., Cardillo R., Mammarella I.C. (2021). Executive functions in neurodevelopmental disorders: Comorbidity overlaps between attention deficit and hyperactivity disorder and specific learning disorders. Front. Hum. Neurosci..

[25] Wilcutt E.G., Petrill S.A. (2023). ADHD and executive function: Insights into cognitive impairment. J. Child. Psychol. Psychiatry.

[26] Pasqualotto A., Fabbro F., Rucco G. (2021). Cognitive rigidity in autism: Implications for adaptation and behavior. Autism Res..

[27] Zelazo P.D. (2022). Executive function: Theory and assessment. Dev. Rev..

[28] Al-Yagon M., Edelstein V., Elder Z. (2020). Social difficulties and executive functions: The hidden link in learning disabilities. J. Spec. Educ..

[29] García Carmona I., Lejárraga García A., Sánchez Sánchez N., de la Cueva M., Díaz Palencia J.L. (2024). Revisión del estado sobre las dificultades en el aprendizaje de las matemáticas en alumnado con TEA. REIDOCREA.

[30] Gioia G.A., Isquith P.K., Guy S.C., Kenworthy L. (2016). Behavior Rating Inventory of Executive Function (BRIEF-2).

[31] Pina-Paredes V., Hernández-Pérez E., Rabadán-Rubio J., Hernández-Pallerés E., Fenollar-Cortés J. (2020). PREDISCAL. Screening de Dificultades Lectoras y Matemáticas.

[32] Saied B. (2022). Executive functions, gender, and personality traits in students with and without specific learning disabilities. Educ. Res. Rev..

[33] García T., Rodríguez C., Castro P.G., Álvarez D., Cueli M., Pienda J.A.G. (2013). Funciones ejecutivas en niños y adolescentes con trastorno por déficit de atención con hiperactividad y dificultades lectoras. Int. J. Psychol. Psychol. Ther..

[34] Paccaud A., Keller R., Luder R., Pastore G., Kunz A. (2021). Satisfaction with the collaboration between families and schools–the parent’s view. Front. Educ..

